# Does performance-based financing increase value for money in low- and middle- income countries? A systematic review

**DOI:** 10.1186/s13561-016-0103-9

**Published:** 2016-07-29

**Authors:** Anne-Marie Turcotte-Tremblay, Jessica Spagnolo, Manuela De Allegri, Valéry Ridde

**Affiliations:** 1 University of Montreal Public Health Research Institute, 7101 Avenue du Parc, office 3060, Montreal, QC Canada H3N 1X9; 2 School of Public Health, University of Montreal, 7101 Avenue du Parc, Montreal, QC Canada H3N 1X9; 3 Douglas Mental Health University Institute, 6875 LaSalle Blvd., Montreal, QC Canada H4H 1R3; 4 Institute of Public Health, Medical Faculty, Heidelberg University, Im Neuenheimer Feld 324, 69120 Heidelberg, Germany

**Keywords:** Performance-based financing (PBF), Economic evaluation, Efficiency, Low- and middle- income countries (LMICs), Review

## Abstract

Governments of low- and middle-income countries (LMICs) are widely implementing performance-based financing (PBF) to improve healthcare services. However, it is unclear whether PBF provides good value for money compared to status quo or other interventions aimed at strengthening the healthcare system in LMICs. The objective of this systematic review is to identify and synthesize the existing literature that examines whether PBF represents an efficient manner of investing resources. We considered PBF to be efficient when improved care quality or quantity was achieved with equal or lower costs, or alternatively, when the same quality of care was achieved using less financial resources. A manual search of the reference lists of two recent systematic reviews on economic evaluations of PBF was conducted to identify articles that met our inclusion and exclusion criteria. Subsequently, a search strategy was developed with the help of a librarian. The following databases and search engines were used: PubMed, EconLit, Google Scholar and Google. Experts on economic evaluations were consulted for validation of the selected studies. A total of seven articles from five LMICs were selected for this review. We found the overall strength of the evidence to be weak. None of the articles were full economic evaluations; they did not make clear connections between the costs and effects of PBF. Only one study reported using a randomized controlled trial, but issues with the randomization procedure were reported. Important alternative interventions to strengthen the capacities of the healthcare system have not been considered. Few studies examined the costs and consequences of PBF in the long term. Important costs and consequences were omitted from the evaluations. Few LMICs are represented in the literature, despite wide implementation. Lastly, most articles had at least one author employed by an organization involved in the implementation of PBF, thereby resulting in potential conflicts of interest. Stronger empirical evidence on whether PBF represents good value for money in LMICs is needed.

## Introduction

Governments and international organizations are investing resources to reduce preventable deaths and diseases across low-and middle- income countries (LMICs). Still, the World Health Organization (WHO) [1] reports that between 20–40 % of resources spent on health are being wasted. Inefficiency is caused by inappropriate use of medicine and equipment, medical errors, suboptimal quality of care, costly staff mix, unmotivated healthcare workers, and corruption [1]. Faced with these issues, program planners must make difficult decisions about the best ways to invest limited resources to improve healthcare services and population health.

In recent years, many governments, donors, consultancy firms and non-governmental organisations (NGOs) have started transforming the funding mechanisms of healthcare systems in LMICs, namely by implementing performance-based financing (PBF) to link payments to results. In this model, healthcare facilities are paid based on the extent to which providers meet pre-defined quantity- and quality-related performance targets, following an independent verification [2]. Examples of quantity-related performance indicators include the number of consultations for children under the age of five or the number of births per month. Examples of quality-related performance indicators include the healthcare center’s cleanliness or completeness of patient registries. Healthcare centers sometime have to reach a minimal quality score (e.g., at least 50 %) in order to be eligible for bonuses. Quality scores are also used as an inflator or deflator of bonus payments.

The implementation of PBF is rapidly expanding. For example, the World Bank reports that the number of African countries using PBF increased from four to 21 between 2006 and 2013 [3]. Despite the rapid implementation of PBF, it is unclear whether given the same amount of resources, PBF can buy more healthcare services or health than the status quo or other interventions aiming to strengthen the healthcare system in LMICs. Existing systematic reviews on economic evaluations of PBF mainly draw their conclusions from studies conducted in high-income countries (HICs) [4, 5]. The results of these systematic reviews therefore cannot be generalized to LMICs, seeing that contexts and resources differ significantly. Distinctive characteristics of LMICs may influence the relations between the costs of PBF and the outcomes observed in HICs. For instance, the initial fixed costs associated with building data infrastructure or monitoring systems may require different investments. According to Fritsche et al. [3], PBF programs tend to require about five percent of additional financing in Organization for Economic Cooperation and Development (OECD) countries compared to 30–40 % of additional financing in LMICs. Moreover, factors unrelated to the motivation of health workers or outside of their control may affect healthcare services to a greater degree in LMICs compared to HICs. On the provider side, these factors may be related to the lack of continuous training, drug supplies, tools and the availability of other resources. On the service-user side, these factors can be related to the difficulty of paying direct and indirect user-fees [6, 7]. Thus, it is important to evaluate whether PBF represents good value for money specifically within the context of LMICs.

The objective of this systematic review is to identify and synthesize the existing literature that examines whether PBF represents an efficient manner of investing resources. In line with Emmert et al.’s approach [4], pay-for-performance (P4P) was considered efficient when improved care quality or quantity is achieved with equal or lower costs, or alternatively, when the same quality or quantity of care was achieved using less financial resources.

## Review

### Methods

#### Protocol and registration

We conducted a systematic review to identify and synthesize literature on economic evaluations of PBF in LMICs. This review is in line with the PRISMA statement [8]. The initial protocol was not registered.

#### Eligibility criteria

##### *Inclusion criteria*

In this systematic review, we included: 1) studies conducted in LMICs, as define by the World Bank [9]; 2) studies using experimental or observational designs to assess the costs (or inputs) and consequences (or outputs); and 3) studies in which a comparison between alternatives was made (including the status quo). We included studies that were primarily impact evaluations only if they also presented results on the costs of PBF. Following Drummond et al.’s [10] categorization scheme, we differentiated studies depending on whether costs, consequences, or both were considered. This approach results in a classification that distinguishes: “Type I” studies as full economic evaluations that make a clear connection between the costs and consequences of two or more alternatives (e.g., cost-effectiveness analyses, cost-utility analyses or cost-benefit analyses); “Type II” studies as partial economic evaluations that describe the costs and consequences of initiatives without making a clear connection between the two; “Type III” studies that compare the costs of the initiatives without providing an effectiveness analysis regarding the health services or health outcomes; and “Type IV” studies that provide information on the costs of a PBF initiative without any description of changes in healthcare services or health outcomes [4]. To avoid overlooking important literature, we included articles belonging to these four types of economic evaluation studies.

##### *Exclusion criteria*

In this systematic review, we excluded: 1) studies conducted in HICs, as defined by the World Bank [9]; 2) publications that did not provide empirical evidence, such as editorials and interviews; 3) non-comparative evaluations because full economic evaluations require the comparison of two alternatives; 4) studies that only described a PBF program or solely evaluated their effectiveness; and 5) studies that focused only on demand-side financial incentives, such as financial compensations or bonuses for people who seek healthcare.

#### Information sources

##### *Searching in previous systematic review*

We began our search by manually screening the reference lists of two recent systematic reviews to find economic evaluations of PBF focusing specifically on LMICs. A well-cited review, conducted by Emmert et al. [4], covered economic evaluations of PBF published between January 2000 and April 2010. The authors did not impose location-related restrictions. Meacock et al. [5] repeated the same search in September 2012 to ensure that no recent articles were omitted. We also screened the reference lists of additional relevant reviews that came to our attention during the search [11–14]. By reviewing past systematic reviews, we were able to identify pertinent studies published between January 2000 and September 2012.

##### *Searching in databases*

As Rethlefsen et al. [15] recommend, we collaborated with a professional librarian from the University of Montreal. We adapted Emmert et al.’s [4] search strategy to find more recent literature on economic evaluations of PBF in LMICs. Our search differed from Emmert et al. [4]'s in that we: 1) added Mesh terms and descriptors to expand the search; 2) modified the list of search terms by using more truncated terms (e.g., “cost*” includes “cost-effectiveness”); 3) deleted currency-related terms (e.g., dollars, yen) to better target pertinent results, given the rapid expansion of PBF worldwide; and 4) updated the inclusion and exclusion criteria (see below).

We conducted electronic searches in two databases: PubMed and Econlit. Search limits included studies written in English and French, published between January 2012 and June 2014. These dates allowed us to have an overlap with the time frame covered by previous systematic reviews to avoid missing any pertinent articles [5]. The complete search history is available in Appendix 1.

In addition to the two databases listed above, we used Google and Google Scholar to identify other potentially relevant documents such as books, unpublished studies, study protocols, conference articles, and new PBF initiatives. We consulted the websites of governmental and scientific institutes concerned with PBF (e.g., the World Bank's website on results-based financing, www.rbfhealth.org; the Global Fund, http://www.theglobalfund.org). We also contacted health economics experts to request information on additional ongoing or recently completed studies. We provided them with a list of the articles selected for this review and invited them to identify any missing article.

#### Study selection

One investigator judged titles and abstracts of potentially relevant studies according to inclusion and exclusion criteria (Table 1). When the investigator could not reach a final decision based on the abstract solely, she proceeded to review the full text. If a decision was still unattainable, a second investigator reviewed the article before reaching a consensual decision. Two investigators read and appraised the articles selected.

**Table 1:** Inclusion and exclusion criteria

	Inclusion criteria	Exclusion criteria
Language	English, French	Other languages
Publication type	All documents presenting empirical data (e.g., peer-reviewed articles)	Protocols, editorials, guidelines and interviews
Study type	Experimental or observational studies including a quantitative assessment of 1) costs and effects, or 2) costs alone	Qualitative studies or studies that only examine effects
Economic evaluation type	Comparative evaluations: full economic evaluations and partial economic evaluations	Non-comparative evaluations
Targeted entity	Healthcare providers	Solely patients
Country	LMICs	HICs

#### Data items and extraction

Two members of the research team performed data extraction. The data extraction forms were custom-designed. The following information was extracted to summarize the articles: first author, publication year, country where study was conducted, characteristics of the PBF program, study objective (implicit or explicit), sample size, data gathering techniques, primary data analysis approach and main results of the study in relation to our focus.

#### Summary measures and data synthesis

The studies selected used a variety of principal summary measures (e.g., technical efficiency scores, Malmquist Productivity Index, difference in costs). Where possible, we present the effects of the interventions as the difference between the intervention and control groups at baseline and follow up percentages or scores. We could not perform a meta-analysis due to heterogeneity of studies and presentation of results.

#### Appraising methodological and reporting quality of included studies

We appraised the results of the studies by examining the relation established between the costs and consequences; the alternative interventions that were considered; the costs and consequences that were included or omitted; the study limitations; and potential conflicts of interests.

To help us synthesize our assessment of the overall strength of the evidence, we developed a concise list of questions, adapted from Drummond et al. [10].

1. Was a clear relation between costs and consequences demonstrated empirically?
2. Which types of designs were used to assess the effectiveness of PBF?
3. Were different types of interventions considered as alternatives?
4. Were the costs (or inputs) and consequences measured longitudinally to examine change over time?
5. Were all important costs (or inputs) and consequences considered?
6. Were the studies conducted in different countries and contexts?
7. Did the authors report potential conflicts of interest?

## Results

### Study selection

**Fig. 1:**
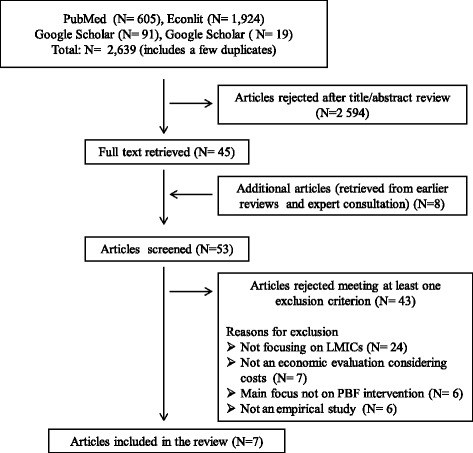
Search flow and results

In total, we identified 2, 639 potentially relevant articles throughout PubMed, Econlit, Google Scholar and Google. After eliminating duplicates and reviewing the remaining abstracts, 45 studies were retained for a more detailed analysis. Screening reference lists from earlier reviews and expert consultations yielded 8 additional articles. Thus, 53 full texts were assessed. Of these, seven studies met our inclusion criteria (Fig. 1) and were included in the review.

Appendix 3 presents a list of articles that were screened, but then excluded. The most common reason for exclusion was that the articles did not focus on LMICs.

### Study characteristics and appraisal

We present a summary of each study’s characteristics in Table 2. The table highlights the diversity of intervention designs, study methods and outcomes. We also provide a summary of our appraisal for each study in Table 3.

**Table 2:** Characteristics of included studies

Author (year) Country	PBF program	Objectives	Sample	Data gathering	Data analysis	Main results
Bowser (2013) [19] Belize	National Health Insurance (NHI) using performance contracts. Implemented in 2001. Expanded in 2006.	To assess trends in financial sustainability, efficiency payments, bonuses and health system and health outcomes.	*Contracted facility areas* : 3 private, 5 public	Data obtained from databases at the facility, district and national levels.	Descriptive trend analysis.	Per capita spending on health services provided by the NHI program decreased from approximately BZ$177 (i.e., US$ 89) to BZ$ 136 (i.e., US$ 68) between 2006 and 2009.
			*Non-contracted facility areas*: providers in three districts financed by the MOH.		Difference-in-difference approach (technical efficiency indicators).	NIH-contracted facility areas had greater improvements in facility births, nurse density, reducing maternal mortality, diabetes deaths, and morbidity compared to non-contracted areas. However, NIH-contracted facility areas had worst outcomes for physician density and death per hypertension between 2006 and 2010.
Gok (2015) [18] Turkey	Pay-for-performance (P4P) program implemented in public and private hospitals implemented since 2004.	To analyze the effects of the P4P system on the hospitals’ efficiencies.	251 hospitals of which 25 are private and 226 are public.	Data obtained from the Annual Statistical Health Report (2001–2008) and the Statistical Institute.	Data envelopment analysis (technical efficiency scores).	In public hospitals, the average efficiencies increased from 0.68 in 2005 to 0.73 in 2008, after the P4P system was adopted. In private hospitals, the average efficiencies decreased from 0.75 in 2005 to 0.61 in 2008.
					Productivity trends (Malmquist Productivity Index).	In public hospitals, the efficiency trend increased from 0.981 in the pre-P4P period to 1.018 after the implementation of the PFP system. In private hospitals, the efficiency trend decreased from 1.016 in the pre-PFP period to 0.967 after the implementation of the P4P system.
Zeng (2013) [21] Haiti	PBF program initiated in 1999 and scaled-up in 2005. Funded by USAID.	To evaluate the costs of implementation as well as the impact of PBF and/or international support (training & monitoring) on primary healthcare services.	15 health centers with PBF and 202 without PBF.	Routine data on the quantity of services provided & 12 interviews with NGO and health facility management staff.	Difference-in-differences approach (growth of incentivized vs non-incentivized services).	Incentive payments added 6 % to base costs of PBF while international support added 39 %. Incentives alone were associated with a 39 % increase in health services. Support alone was associated with a 35 % increase in health services. Support combined with incentives was associated with an 87 % increase compared with health facilities that did not receive either. Non-incentivized services did not perform significantly lower than incentivized services.
Basinga (2011) [16] Rwanda	P4P scheme providing incentives to providers for improvements in utilisation and quality of care. National program gradually implemented since 2005, after pilot schemes by NGOs.	Assess the effect of P4P on the use and quality of child and maternal care services.	80 health facilities were assigned to a P4P program and 86 health facilities were assigned to be control facilities. 2 158 households were also included.	Facilities and households were surveyed at baseline and after 23 months.	Descriptive statistics from annual reports at the national level.	The administrative costs associated with P4P were estimated to be US$ 0.3 per person in total, representing 0.8 % of total health expenditures per person and 1.2 % of public and donor expenditures combined.
					Difference-in-difference model (multivariate regression).	The intervention group had a 23 % increase in institutional deliveries, a 56 % increase in preventive care visits by children aged 23 months or younger, and a 132 % increase in preventative care visits by children between 24 and 59 months, compared to the control group. However, there were no improvement in the number of women receiving any prenatal care, the number of women completing four or more prenatal visits, and the number of children receiving full immunisation schedules.
Rusa (2009) [22] Rwanda	PBF (reimbursement mechanism with ‘indicator purchasing’ linked to formative supervision). Implemented in 2005. Expanded in 2006. Funded by the Belgian Cooperation.	To evaluate the effect of PBF on the performance of healthcare centers.	74 health centers that implemented PBF in 2005 and 85 health centers that implemented PBF in 2006.	Data on services were collected on a monthly basis by the district supervisors.	Time-series with a two-staged implementation but only descriptive statistics.	The part of the subsidies spent on the functioning of the health facility, grew from approximately 8 % in 2005, to 23 % in 2006 and to 38 % in 2007. Overall, the budget allocated to the implementation of a PBF program amounted to US$ 0.25/cap/year, of which US$ 0.20/cap/year for subsidies and an estimated US$ 0.05/cap/year for administration, supervision and training. Results showed a positive effect for activities that were less organized (i.e., monitoring services and institutional deliveries). No effects were found on curative consultations, family planning, antenatal consultations and vaccinations. Compliance rates with norms rose in both groups.
Sabri (2007) [20] Afghanistan	3 NGO contracting programs with capitation payments to providers for each individual enrolled. Implemented since 2001. Funded by World Bank, USAID or European Commission.	To analyze the financing and costs of contracting healthcare services.	No description provided.	Statistics from government and NGO reports.	Descriptive statistics.	The reference cost used to negotiate the delivery of a basic package of health services with contracted NGOs was estimated to be US$ 4.5 for 2002. The cost varied among the different donors. The annual per-capita cost was US$ 3.8 for the World Bank, US$ 4.2 for USAID and US$ 5.1 for the European Commission. The population coverage for basic health services increased from 9 % in 2002 to 82 % in 2006. However, the quality of services provided appeared to be poor (e.g.,: long waiting times, absence of laboratory services, shortage of drugs, and disrespect for patients). Facilities run under the ministry's strengthening mechanism and NGO contracts under the World Bank and the USAID performed better than contracts held by the European Commission due to cumbersome administrative procedures. Authors discuss the preliminary results of an Afghanistan household survey suggesting that under five child and infant mortality rates improved.
Soeters (2006) [23] Rwanda	P4P program introduced in 2002 by Cordaid.	To present Rwanda’s P4P experience.	240 and 320 households in province with P4P.	Household surveys in 2003 and 2005.	Difference-in-difference approach (no clear description of analyses).	Out-of-pocket health expenditure decreased by 62 %, from US$ 9.05 to US$ 3.45. The percentage of respondents who experienced a catastrophic user fee payments decreased from 2.5 % in 2003 to 0.7 % in 2005. The proportion of women delivering in a health facility increased from 25 % to 60 %. In the discussion, authors indicate that the administrative costs of the fundholder were about 25 % of the total contracting costs, according to Cordaid data.

**Table 3:** Appraisal of included studies

Author (year)	Was a clear relation between costs & consequences established?	Which alternative intervention was considered?	The costs (or inputs) and consequences were measured over which time frame?	Were important costs (or inputs) and consequences omitted?	What were the main limitations?	Are there potential conflicts of interest?
Bowser (2013) [19]	No	Status quo (traditional salaries and line-item budgets).	2006 to 2010	No clear description of the included and omitted costs.	1) absence of pre-intervention data; 2) possibility that other factors influenced the costs per capita; 3) difficulty of teasing apart the effects due to the incentives from those related to other components of the reform.	- None declared - 1 author affiliated with the organisation involved in the implementation
Gok (2015) [18]	No	Status quo (before vs after P4P).	2001-2008	Yes, for example, the costs of implementing the program were not included.	1) the absence of randomization; 2) the lack of a control group; and 3) the use of aggregate input and output variables.	- None declared
Zeng (2013) [21]	No	International support (including procurement procedures, minor renovations, advice on community mobilization, communication, public relations & promotion of family planning).	2008-2010	No clear description of the included and omitted costs for the "international support".	1) absence of randomization; 2) the absence of pre-intervention data; 3) the lack of control for the quality of the data in the 202 health centers without PBF.	- Declaration that one co-author was employed by an organisation involved in PBF.
Basinga (2011) [16]	No	Input-based budgets in the control group were increased by the average P4P payments made to the intervention group	June, 2006 to Avril 2008 (~23 months)	Lack of detailed information on the costs of PBF. Health outcomes were not included.	1) the absence of pre-intervention data; 2) problems identified with allocation to treatment and control-groups (see Witter et al., 2013).	- None declared - Some authors affiliated with organisations involved in the funding and implementation.
Rusa (2009) [22]	No	Status quo for performance data (3 months of pre-intervention data). No alternative intervention was used to compare costs.	2005 to 2007 for costs. October 2014 to December 2007 for performance.	Includes subsidies and administration costs. No detailed description of the included and omitted costs. Health outcomes were not included.	1) insufficient use of pre-intervention data; 2) the lack of a control group without PBF during the entire time period; and 3) the possibility that other interventions (e.g., mutual health organizations, sensitization campaigns) influenced the results.	- None declared - 5 of 6 authors affiliated with organisations involved in the implementation
Sabri (2007) [20]	No	Comparison of three different PBF programs.	2002 to 2006	No clear description of the included and omitted costs. Limited data on healthcare services and health outcomes.	1) the lack of information on the methodology used; and 2) the absence of links between the costs and outcomes.	- None declared - At least one author employed by an organisation involved in the implementation
Soeters (2006) [23]	No	Comparison of PBF in early vs later stages.	2003 to 2005	Lack information on how PBF affects total health expenditures (only focuses on out-of-pocket health spending).	1) absence of pre-intervention data; 2) absence of a control group; and 3) possibility that other interventions occurring simultaneously reduced catastrophic user fee payments.	- None declared - Authors worked for an organisation involved in the implementation

### Synthesis of results and appraisal

The section below presents our overall assessment of the strength of the evidence, using the list of questions we adapted from Drummond et al. [10].

#### 1. Was a clear relation between costs and consequences demonstrated empirically?

None of the included studies were classified as full economic evaluations that make clear connexions between the PBF costs and healthcare services and/or health (Type I). In other words, none of the studies included cost-effectiveness analyses, cost-utility analyses or cost-benefit analyses. For this reason, we classified the 7 studies as partial economic evaluations (Type II), as they described the costs and consequences of PBF initiatives without making a clear connection between the two. It is important to note that full economic evaluations are necessary to evaluate whether PBF provides good value for money in LMICs because they are more methodologically sound than partial economic evaluations [10].

#### 2. Which types of designs were used to assess the effectiveness of PBF?

An intervention that is not effective cannot provide good value for money. Therefore, we examined the designs that were used to assess the effectiveness of PBF in the included studies. Of the seven articles, only one study reported using a randomized control trial to assess the consequences of PBF [16]. However, Witter and colleagues [14] have identified problems with the allocation to the treatment and control-groups for this study. It appears that some districts were found to have existing pay for performance schemes, requiring the allocation to be adjusted in a non-random way. This study found that the intervention group had an increase in institutional deliveries and preventive care visits, compared to the control group. However, there was no improvement in the number of women receiving any prenatal care; the number of women completing four or more prenatal visits; and the number of children receiving full immunisation schedules.^1^ The other articles included in this review adopted a variety of observational designs, for instance, relying on difference-in-difference estimates, time series and trend analyses. The majority of studies did not use pre-intervention data in their analyses. Potential biases and mitigated results limit our confidence in the effectiveness of PBF programs, as presented in the studies.

#### 3. Were different types of interventions considered as alternatives?

Economic evaluations require the comparison of two alternatives to identify which is more efficient [2]. Most studies in this review compared the implementation of PBF to the status quo. System-strengthening alternatives to improve the motivation of healthcare workers or service delivery were not used as comparators. Potential alternatives that could have been considered to test whether PBF provides the best value for money include: other funding mechanisms; monitoring (without financial incentives); providing performance feedback; training health workers; increasing leadership skills; encouraging collaboration; and fostering a culture that promotes trust and the intrinsic value of work [17]. In addition, more studies should attempt to tease apart the incentive effect from the resource effect. Only one study included in this systematic review increased the budgets of the PBF intervention and control groups by the same amount [16].

#### 4. Were the costs (or inputs) and consequences measured longitudinally to examine change over time?

The seven articles examined the impact of PBF programs over different time periods. Gok & Altmdag [18]’s study ranges from 2001–2008; Bowser et al. [19] and Sabri et al. [20]’s study cover a four-year time period; and Zeng et al. [21], Basinga et al. [16], Rusa et al. [22], and Soeters et al. [23] report change over a two year period. From the studies in this review, little is known about how the relation between PBF costs and outcomes in LMICs evolves over the long term.

#### 5. Were all important costs (or inputs) and consequences considered?

The studies did not provide a detailed description of the costs that were included or omitted. The studies mostly examined the immediate/direct financial costs and effects of the interventions. Authors generally did not attempt or were not able to quantify all the different types of costs and inputs (e.g., time and funds invested to monitor the delivery of health services, time spent filling out forms). Only aggregated costs were presented.

Overall, important effects on health outcomes and unintended consequences (e.g., reduction of healthcare services not rewarded financially) were not sufficiently considered.

#### 6. Were the studies conducted in different countries and contexts?

The seven articles were conducted in only five LMICs. Table 4 presents the number of articles, the region and the income level for each of these countries. Many regions and countries currently implementing PBF are not represented in these studies [24]. Moreover, some countries like Rwanda are characterised by unique political contexts and demographic situations, limiting the generalizability of results to other countries.

**Table 4:** Countries classified according to the region and income level

Country	Number of articles	Region	Income level
Rwanda	3	Sub-Saharan Africa	Low-income economy
Belize	1	Latin America and the Caribbean	Upper middle-income economy
Haiti	1	Latin America and the Caribbean	Low-income economy
Afghanistan	1	South Asia	Upper middle-income economy
Turkey	1	Europe and Central Asia	Upper middle-income economy

^a^This classification is based on World Bank criteria

#### 7. Did the authors report potential conflicts of interest?

Six out of seven articles had at least one author that was or had been affiliated with an organisation involved in the implementation of PBF, thereby resulting in a potential conflict of interest. The interpretation of data or presentation of information may have been influenced by their personal or financial relationship with other people or organizations. Interestingly, only one author explicitly reported having been employed by an organization involved in the implementation of PBF as a potential conflict of interest [21].

#### Summary of the assessment

Only seven articles fit out inclusion criteria. Overall, the evidence of economic evaluations of PBF is weak for the following reasons: (1) none of the studies were full economic evaluations; (2) only one study used a randomized controlled trial, but issues with the randomization procedure were reported; (3) important alternative interventions to strengthen the capacities of the healthcare system have not been used as a comparator; (4) few studies examined the costs and consequences of PBF over the long term; (5) important costs and consequences were omitted from the evaluations; (6) very few LMICs are represented in the literature, despite wide implementation in these countries; and (7) most articles had at least one author that was affiliated with an organisation involved in the implementation of PBF, thereby resulting in a potential conflict of interest.

## Discussion

This systematic review highlights a lack of strong empirical evidence that supports the idea that PBF increases value for money in LMICs. This result is consistent with past findings [4, 5, 11, 14]. For example, a Cochrane review addressing the effectiveness of PBF in LMICs found that the current evidence base is too weak to draw general conclusions about the effectiveness of PBF in LMICs. Without reliable effectiveness-estimates, cost-effectiveness estimates cannot be calculated. Thus, it would have been surprising if this review had concluded differently.

The added value of this review is threefold. First, replications of past reviews are useful to validate results and find articles that might have been overlooked. Second, past reviews only included studies published up to 2011–2012. An update was therefore warranted, especially considering the rapid implementation of PBF in LMICs and the large number of studies that have published on PBF since then. Third, this is the first literature review with a search strategy that specifically targeted articles on the efficiency of PBF in LMICs. Thus, the current review has a different focus than past reviews, providing a collection of economic evaluations of PBF in LMICs that were not previously identified. For example, six of the seven studies in this systematic review were not included in the Cochrane review. Three of the studies were published after the Cochrane authors conducted their search [18, 19, 21]. The three other studies included in this systematic review, but not in the Cochrane review, were published and available in time to be considered [20, 22, 23]. However, they were not included and are not mentioned under “excluded studies” in the Cochrane review. Consequently, our systematic review may be useful to inform researchers and decision-makers specifically concerned with optimizing value for money in LMICs.

The reasons why so few PBF economic evaluations have been conducted in LMICs, despite wide implementation, is worth exploring. First, PBF is a complex intervention that targets multiple services. It is therefore difficult to evaluate the impact of PBF on health. Economic evaluations on this topic require complex modelling because diverse people and many conditions are affected. Second, it is difficult to obtain good quality cost data in LMICs because the information is not easily accessible. Last, international partners occasionally resist sharing their costs, usually substantial at start up. Promoting transparency may be useful to facilitate economic evaluations on PBF.

### Strengths and limitations

While systematic reviews can take years to complete, this review was conducted within a few months to respond to timely concerns about whether PBF provides the best value for money in LMICs. The time frame usually required for producing systematic reviews has been found to be inappropriate for local policy makers that have urgent decisions to make [25]. This issue was highlighted by a decision-maker in Haiti, who widely shared an e-poster on the current results, claiming that “long publication delays would eliminate the important benefits of this review” (personal communication, June 13, 2015). Despite its rapidity, this review adheres to the core principles of systematic reviews in order to avoid bias and ensure rigor. A detailed description of the methods used was provided to promote methodological transparency, and to facilitate replication.

Our review has limitations. First, the studies varied in methodological quality and study characteristics. These differences made it difficult to adequately compare the results of the articles included in our systematic review. Second, as in the case with most reviews, our review might have suffered from publication bias. Sponsors of inefficient PBF programs may have blocked publishing to protect their interests [4]. Last, as with any review, we may have missed some relevant information during the selection and data extraction process.

### Future directions

Future researchers and evaluators should attempt to make a direct relation between costs and consequences of PBF in order to draw conclusions about whether this financing option represents good value for money. There is a need to adopt stronger designs and to consider the long-term implications of these programs on costs and health outcomes. In addition, future studies should compare PBF to promising alternative interventions that aim to strengthen the healthcare system. It would also be beneficial to analyze the literature around PBF in LMICs using Drummond and Jefferson (1996)’s 38 defined quality criteria, as seen in Emmert et al. [4]'s systematic review, in order to generate an average quality score for each article.

During our search, it has come to our attention that at least three economic evaluations of PBF are currently being conducted in LMICs. Borghi et al. [26] published a protocol on the evaluation of a P4P program in Tanzania. Using a controlled before and after study, the authors aim to measure the cost-effectiveness of the P4P program. Moreover, two economic evaluations are being conducted on PBF initiatives in Malawi [27]. Together, these studies should contribute to the evidence on the efficiency of PBF in LMICs.

## Conclusion

In contexts of limited resources such as LMICs, it is essential that funders and decision-makers aim to optimize the value obtained from the money invested in healthcare services, in order to address the pressing health needs of the population. Some stakeholders have proposed PBF as a promising avenue. However, this review has demonstrated that there is a lack of empirical evidence to support the claim that PBF represents good value for money. We still do not know if, given the same amount of resources, PBF buys more healthcare services or health than the status quo or other interventions. Full economic evaluations of PBF are needed to truly inform decision-makers in LMICs on how to make better use of limited resources to improve population health.

## Appendix 1 : Literature Search History

### Appendix 1.1 : PubMed Search History

**#1: 2014/06/20; N = 2079**

Search (((("pay for performance" OR "P4P" OR "PFP" OR "pay for value" OR "pay for quality" OR "payment for quality" OR "value-based purchasing" OR ("financial incentive*" AND quality) OR ("monetary incentive*" AND quality) OR (bonus AND quality) OR (reward* AND quality) OR "performance-based payment" OR "performance-based reimbursement" OR "performance-based contracting" OR "performance-based pay" OR "performance-based financing" OR "results-based financing" OR "output-based payment" OR "incentive reimbursement" OR "incentive program" OR "quality-based purchasing" OR "quality incentive" OR "Quality Improvement/economics"[Mesh] OR "Reimbursement, Incentive/economics"[Mesh] OR "Financing, Government/methods"[Mesh] OR "Quality Assurance, Health Care/economics"[Mesh]))) AND ((French[Language]) OR English[Language])) AND ("2012/01/01"[Date - Publication] : "2014/06/20"[Date - Publication])

**#2: 2014/06/20; N = 157 204**

Search (((("program evaluation*" OR "economic evaluation*" OR "financial analysis" OR "cost*" OR "profit" OR "efficiency" OR "efficient" OR "return on investment" OR "ROI" OR "rate of return" OR "net present value" OR "benefit-cost ratio" OR "business case" OR "economic case" OR "social case" OR "quality-adjusted life years" OR "qaly*" OR "Costs and Cost Analysis/economics"[mesh] OR "Cost Control/economics"[mesh] OR "Cost Savings/economics"[mesh] OR "Cost-Benefit Analysis/economics"[mesh] OR "Program Evaluation/economics"[mesh] OR "Health Services Research/economics"[mesh] OR "Utilization Review/economics"[mesh] OR "Efficiency, Organizational/economics"[mesh]))) AND ((French[Language]) OR English[Language])) AND ("2012/01/01"[Date - Publication]: "2014/06/20"[Date - Publication])

**#3: 2014/06/20; N = 605**

Search #1 AND #2

### Appendix 1.2 Econlit Search History

**#1: 2014/06/20; N = 10,030**

AB (("pay for performance" OR "p4p" OR "pfp" OR "pay for value" OR "pay for quality" OR "payment for quality" OR "value-based purchasing" OR ("financial incentive*" AND "quality") OR ("monetary incentive*" AND "quality") OR ("bonus" AND "quality") OR ("reward*" AND "quality") OR "performance-based pay*" OR "performance-based reimbursement" OR "performance-based contracting" OR "output-based payment" OR "incentive reimbursement" OR "incentive program" OR "quality-based purchasing" OR "quality incentive" OR "performance-based financing" OR "results-based financing")) or TI (("pay for performance" OR "p4p" OR "pfp" OR "pay for value" OR "pay* for quality" OR "value-based purchasing" OR ("financial incentive*" AND "quality") OR ("monetary incentive*" AND "quality") OR ("bonus" AND "quality") OR ("reward*" AND "quality") OR "performance-based payment" OR "performance-based reimbursement" OR "performance-based contracting" OR "performance-based pay*" OR "output-based payment" OR "incentive reimbursement" OR "incentive program" OR "quality-based purchasing" OR "quality incentive" OR "performance-based financing" OR "results-based financing")) or SU (("Analysis of Health Care Markets" OR " Health: Government Policy; Regulation; Public Health" or "National Government Expenditures and Health " or "Economic Development: Human Resources; Human Development; Income Distribution; Migration"))

Dates: January 2012 to July 2014

**#2: 2014/06/20; N = 24,137**

AB (("program evaluation*" OR "economic evaluation*" OR "financial analysis" OR "saving*" OR "cost*" OR "profit" OR "efficiency" OR "efficient" OR "return on investment" OR "roi" OR "rate of return" OR "net present value" OR "business case" OR "economic case" OR "social case" OR "quality-adjusted life years" OR "qaly*")) or TI (("program evaluation*" OR "economic evaluation*" OR "financial analysis" OR "saving*" OR "cost*" OR "profit" OR "efficiency" OR "efficient" OR "return on investment" OR "roi" OR "rate of return" OR "net present value" OR "benefit-cost ratio" OR "business case" OR "economic case" OR "social case" OR "quality-adjusted life years" OR "qaly*"))

Dates: January 2012 to July 2014

**#3: 2014/06/20; N = 1,924**

#1 AND #2

### Appendix 1.3 Google Scholar Search History

**#1: 2014/06/25; N = 91**

("P4P" OR "performance-based financing" OR "results-based financing") AND ("economic evaluation")

("2012"[Date - Publication]: "2014"[Date - Publication])

### Appendix 1.4 Google Search History

**#1: 2014/06/25; N = 19**

("P4P" OR "performance-based financing" OR "results-based financing") AND ("economic evaluation")

("2012/01/01"[Date - Publication]: "2014/06/20"[Date - Publication])

## Appendix 2: Abstracts of included studies

### Appendix 2.1: Bowser et al., 2013

Over the last 10 years, Belize has implemented a National Health Insurance (NHI) program that uses performance-based contracts with both public and private facilities to improve financial sustainability, efficiency and service provision. Data were collected at the facility, district and national levels in order to assess trends in financial sustainability, efficiency payments, yearend bonuses and health system and health outcomes. A difference-indifference approach was used to assess the difference in technical efficiency between private and public facilities. The results show that per capita spending on services provided by the NHI program has decreased over the period 2006–2009 from BZ$177 to BZ$136. The private sector had achieved higher levels of technical efficiency, but lower percentages of efficiency and year-end bonus payments. Districts with contracts through the NHI program showed greater improvements in facility births, nurse density, reducing maternal mortality, diabetes deaths and morbidity from bronchitis, emphysema and asthma than districts without contracts over the period 2006–2010. This preliminary assessment of Belize’s pay-for-performance system provides some positive results, however further research is needed to use the lessons learned from Belize to implement similar reforms in other systems.

### Appendix 2.2: Gok and Altmdag (2015)

This paper analyzes the effects of the pay for performance (PFP) system on the efficiencies of public and private hospitals in Turkey. In order to evaluate these effects, we examine the relationship between hospital efficiency and health care costs in Turkey, and addressed the impact of the PFP system on the efficiencies of public and private hospitals. In an effort to analyze the efficiencies of public and private hospitals, this study used data envelopment analysis. The Malmquist Productivity Index is also used to analyze the patterns of efficiency change for the study years from 2001 to 2008. This study shows that health care costs and hospital efficiency are negatively correlated for private hospitals, while they are positively correlated for public hospitals. In other words, increased health care costs might reduce efficiency in private hospitals in contrast to public hospitals. Our findings also indicate that average efficiencies of public hospitals tend to increase, particularly during the implementation period ofPFP system. The efficiency trend of private hospitals, conversely, decreased in the latter periods of the PFP system. Suggestions for improvement are provided to the health care policy makers regarding the impact of health care reforms on public and private hospitals.

### Appendix 2.3: Zeng et al., 2013

To strengthen Haiti’s primary health care (PHC) system, the country first piloted performance-based financing (PBF) in 1999 and subsequently expanded the approach to most internationally funded non-government organizations. PBF complements support (training and technical assistance). This study evaluates (a) the separate impact of PBF and international support on PHC’s service delivery; (b) the combined impact of PBF and technical assistance on PHC’s service delivery; and (c) the costs of PBF implementation in Haiti. To minimize the risk of facilities neglecting potential non-incentivized services, the incentivized indicators were randomly chosen at the end of each year. We obtained quantities of key services from four departments for 217 health centres (15 with PBF and 202 without) from 2008 through 2010, computed quarterly growth rates and analysed the results using a difference-in-differences approach by comparing the growth of incentivized and non-incentivized services between PBF and non-PBF facilities. To interpret the statistical analyses, we also interviewed staff in four facilities. Whereas international support added 39 % to base costs of PHC, incentive payments added only 6 %. Support alone increased the quantities of PHC services over 3 years by 35 % (2.7 %/quarter). However, support plus incentives increased these amounts by 87 % over 3 years (5.7 %/quarter) compared with facilities with neither input. Incentives alone was associated with a net 39 % increase over this period, and more than doubled the growth of services (P < 0.05). Interview findings found no adverse impacts and, in fact, indicated beneficial impacts on quality. Incentives proved to be a relatively inexpensive, well accepted and very effective complement to support, suggesting that a small amount of money, strategically used, can substantially improve PHC. Haiti’s experience, after more than a decade of use, indicates that incentives are an effective tool to strengthen PHC.

### Appendix 2.4: Basinga et al., 2011

*Background*: Evidence about the best methods with which to accelerate programs towards achieving the Millennium Development Goals is urgently needed. We assessed the effect of performance-based payment of health-care providers (payment for performance; P4P) on use and quality of child and maternal care services in health-care facilities in Rwanda.

*Methods*: 166 facilities were randomly assigned at the district level either to begin P4P funding between June, 2006, and October, 2006 (intervention group; *n* = 80), or to continue with the traditional input-based funding until 23 months after study baseline (control group; *n* = 86). Randomisation was done by coin toss. We surveyed facilities and 2158 households at baseline and after 23 months. The main outcome measures were prenatal care visits and institutional deliveries, quality of prenatal care, and child preventive care visits and immunisation. We isolated the incentive effect from the resource eff ect by increasing comparison facilities’ input-based budgets by the average P4P payments made to the treatment facilities. We estimated a multivariate regression specification of the difference-in-difference model in which an individual’s outcome is regressed against a dummy variable, indicating whether the facility received P4P that year, a facility-fixed effect, a year indicator, and a series of individual and household characteristics.

*Findings*: Our model estimated that facilities in the intervention group had a 23 % increase in the number of institutional deliveries and increases in the number of preventive care visits by children aged 23 months or younger (56 %) and aged between 24 months and 59 months (132 %). No improvements were seen in the number of women completing four prenatal care visits or of children receiving full immunisation schedules. The authors also estimated an increase of 0 · 157 standard deviations (95 % CI 0 · 026–0 · 289) in prenatal quality as measured by compliance with Rwandan prenatal care clinical practice guidelines.

*Interpretation*: The P4P scheme in Rwanda had the greatest effect on those services that had the highest payment rates and needed the least effort from the service provider. P4P financial performance incentives can improve both the use and quality of maternal and child health services, and could be a useful intervention to accelerate progress towards Millennium Development Goals for maternal and child health.

### Appendix 2.5: Rusa et al., 2009

In 2005, the Ministry of Health in Rwanda, with the support of the Belgian Technical Cooperation, launched a strategy of performance-based financing (PBF) in a group of 74 health centres (HCs), covering 2-m inhabitants. In 2006, PBF was extended to an additional group of 85 HCs, thus reaching 3.8-m inhabitants. This study evaluates the effect of PBF on HC performance from 2005 to 2007. Composite indicators for measuring quantity and quality of services were developed and evaluated through monthly formative supervisions by qualified and well-trained district supervisors. The strategy was based on a fixed fee per quality-approved service. The entire budget spent on the implementation of PBF amounted to $0.25 ⁄ cap ⁄ year, of which $0.20 ⁄ cap ⁄ year for subsidies and an estimated $0.05 ⁄ cap ⁄ year for administration, supervision and training. A positive effect on utilization rates was only seen for activities that were previously less well organized; in this case, growth monitoring services and institutional deliveries. The quality of services, defined as the compliance rate with national and international norms, rose considerably for all services in both groups. A sustained level of quality between 80 % and 95 % was reached within 18 months in the first group. A similar result was reached in the second group in 8 months.

### Appendix 2.6: Sabri et al., 2007

Disruption caused by decades of war and civil strife in Afghanistan has led many international and national nongovernmental organizations (NGOs) to assume responsibility for the delivery of health services through contracts with donor agencies. Recently the Afghan Government has pursued the policy of contracting for a basic package of health services (BPHS) supported by funds from three major donors – the World Bank, the United States Agency for International Development (USAID) and the European Commission. With the gradual strengthening of the public health ministry, options for the future include pursuing the contracting option or increasing public provision of health services.

Should contracting with NGOs be pursued, a clear strategy is required that includes developing accreditation instruments, better contracting mechanisms and a system for monitoring and evaluating the entire process. Should the government opt for an increasing role, problems to be solved include securing the transition to public provision, obtaining guarantees that appropriate financing will be provided and reconfiguration of the public health delivery system. Large-scale contracting with the private for-profit sector cannot be recommended at this stage, although this option could be explored via subcontracting by larger NGOs or smallscale trial contracts initiated by the public health ministry. Irrespective of the option chosen, an important challenge remaining is the recalcitrant problem of high out-of-pocket payments.

Sustainable delivery of health services in Afghanistan can only be achieved with a clear national strategy in which all stakeholders have roles to play in the financing, regulation and delivery of services.

### Appendix 2.7: Soeters et al., 2006

Evidence from low-income Asian countries shows that performance-based financing (as a specific form of contracting) can improve health service delivery more successfully than traditional input financing mechanisms. We report a field experience from Rwanda demonstrating that performance-based financing is a feasible strategy in sub-Saharan Africa too. Performance-based financing requires at least one new actor, an independent well equipped fundholder organization in the district health system separating the purchasing, service delivery as well as regulatory roles of local health authorities from the technical role of contract negotiation and fund disbursement. In Rwanda, local community groups, through patient surveys, verified the performance of health facilities and monitored consumer satisfaction. A precondition for the success of performance based financing is that authorities must respect the autonomous management of health facilities competing for public subsidies. These changes are an opportunity to redistribute roles within the health district in a more transparent and efficient fashion.

## Appendix 3

**Table 5:** Characteristics of excluded studies

#	Authors (year)	Reason for exclusion^a^
1	Agee & Gates (2012)	Not focused on LMIC
2	Allen, Nobel & Burton (2012)	Not focused on LMIC
3	Awoonor-Williams (2013)	Main focus not on PBF intervention
4	Baral (2012)	Not an economic evaluation
5	Bernstein (2014)	Not an empirical study
6	Blecker (2014)	Not focused on LMIC
7	Blumenthal, Song, Jena & Ferris (2013)	Not focused on LMIC
8	Broughton et al. (2013)	Main focus not on PBF intervention
9	Chee (2003)	Main focus not on PBF intervention
10	Cheng, Lee & Chen (2012)	Not focused on LMIC
11	Eichler et al. (2013)	Not an empirical study
12	Falisse, Meessen, Ndayishimiye & Brossuyt (2012)	Not an economic evaluation
13	Gerber-Grote & Windeler (2014)	Main focus not on PBF intervention
14	Ginsburg (2013)	Not focused on LMIC
15	Greene, Hibbard & Overton (2014)	Not focused on LMIC
16	Higgs,Stammer, Roth & Balster (2013)	Not an empirical study
17	Himmelstein, Ariely & Woolhandler (2014)	Not focused on LMIC
18	Holcombe (2014)	Not focused on LMIC
19	Hupp (2014)	Not focused on LMIC
20	Ireland, Paul & Dujardin (2011)	Not an empirical study
21	Jeong (2012)	Not an economic evaluation
22	Johnson & Higgins (2014)	Not an empirical study
23	Karash (2013)	Not an empirical study
24	Lee, Cheng, Chen & Lai (2010)	Not focused on LMIC
25	Lorincz, Lawson & Long (2013)	Not focused on LMIC
26	Maynard (2011)	Not focused on LMIC
27	McMahon & Chopra (2012)	Not focused on LMIC
28	Moore & DeBuono (2013)	Not focused on LMIC
29	Peabody et al. (2010)	Main focus not on PBF intervention
30	Rajkumar, Conway & Tavenner (2014)	Not focused on LMIC
31	Ran, Luo, Wu, Yao & Feng (2013)	Not an economic evaluation
32	Robeznieks (2012)	Not an economic evaluation
33	Rosenau, Lal & Lako (2012)	Not focused on LMIC
34	Ryan (2013)	Not focused on LMIC
35	Saronga et al. (2014)	Main focus not on PBF intervention
36	Swensen, Dilling, Mc Carty, Bolton & Harper (2013)	Not focused on LMIC
37	Tan, Pwu, Chen & Yang (2014)	Not focused on LMIC
38	Tummers, Schrijvers & Visser-Meily (2013)	Not focused on LMIC
39	VanLare, Blum & Conway (2012)	Not focused on LMIC
40	Wilson (2013)	Not focused on LMIC
41	Wranik (2012)	Not focused on LMIC
42	Yip et al. (2014)	Not an economic evaluation
43	Zeng, Rwiyereka, Amico, Avila-Figueroa & Shepard (2014)	Not an economic evaluation

^a^ Articles that were excluded often did not meet more than one inclusion criteria. However, only one reason for exclusion is mentioned in the table above

References for excluded studies

Agee, M. D., & Gates, Z. (2012). Lessons from Game Theory about Healthcare System Price Inflation. Applied Health Economics and Health Policy, 11(1), 45–51. http://doi.org/10.1007/s40258-012-0003-z

Allen, H., Nobel, J. J., & Burton, W. N. (2012). Making health care reform work: where physician and employer interests converge. Journal of the American Medical Association, 308(23), 2465–2466. http://doi.org/10.1001/jama.2012.45428

Awoonor-Williams, J. K., Bawah, A. A., Nyonator, F. K., Asuru, R., Oduro, A., Ofosu, A., & Phillips, J. F. (2013). The Ghana essential health interventions program: a plausibility trial of the impact of health systems strengthening on maternal & child survival. BMC Health Services Research, 13 Suppl 2, S3.

Baral, G. (2012). An assessment of the safe delivery incentive program at a tertiary level hospital in Nepal. Journal of Nepal Health Research Council, 10(21), 118–124.

Bernstein, J. (2014). Not by bread alone: shortcomings of the pay-for-performance approach. Clinical Orthopaedics and Related Research, 472(2), 405–409. http://doi.org/10.1007/s11999-013-3417-5

Blecker, E. (2014). The challenges in achieving successful P4P programs. Findings Brief: Health Care Financing & Organization, 42(2), 1–3.

Blumenthal, D. M., Song, Z., Jena, A. B., & Ferris, T. G. (2013). Guidance for structuring team-based incentives in healthcare. The American Journal of Managed Care, 19(2), e64–70.

Broughton, E., Saley, Z., Boucar, M., Alagane, D., Hill, K., Marafa, A., … Sani, K. (2013). Cost-effectiveness of a quality improvement collaborative for obstetric and newborn care in Niger. International Journal of Health Care Quality Assurance, 26(3), 250–261. http://doi.org/10.1108/09526861311311436

Chee, G. (2003). Using financing to motivate a for-profit health care provider to deliver family planning services: Is it a cost-effective intervention? A study of AAR health services in Kenya. The International Journal of Health Planning and Management, pp. 205 – 220.

Cheng, S.-H., Lee, T.-T., & Chen, C.-C. (2012). A longitudinal examination of a pay-for-performance program for diabetes care: evidence from a natural experiment. Medical Care, 50(2), 109–116. http://doi.org/10.1097/MLR.0b013e31822d5d36

Eichler, R., Agarwal, K., Askew, I., Iriarte, E., Morgan, L., & Watson, J. (2013). Performance-based incentives to improve health status of mothers and newborns: what does the evidence show? Journal of Health, Population, and Nutrition, 31(4 Suppl 2), 36–47.

Falisse, J.-B., Meessen, B., Ndayishimiye, J., & Brossuyt, M. (2012). Community participation and voice mechanismes under performance-based financing schemes in Burundi. Tropical Medicine and International Health, pp. 674–682.

Gerber-Grote, A., & Windeler, J. (2014). What is the contribution of health economic evaluations to decision-making in health care? Experiences from 7 selected countries. Zeitschrift Für Evidenz, Fortbildung Und Qualität Im Gesundheitswesen, 108(7), 358–359. http://doi.org/10.1016/j.zefq.2014.08.018

Ginsburg, P. B. (2013). Achieving Health Care Cost Containment Through Provider Payment Reform That Engages Patients And Providers. Health Affairs, 32(5), 929–934. http://doi.org/10.1377/hlthaff.2012.1007

Greene, J., Hibbard, J. H., & Overton, V. (2014). A Case Study of a Team-Based, Quality-Focused Compensation Model for Primary Care Providers. Medical Care Research and Review, 71(3), 207–223. http://doi.org/10.1177/1077558713506749

Higgs, E. S., Stammer, E., Roth, R., & Balster, R. L. (2013). Evidence acquisition and evaluation for evidence summit on enhancing provision and use of maternal health services through financial incentives. Journal of Health, Population, and Nutrition, 31(4 Suppl 2), 23–35.

Himmelstein, D. U., Ariely, D., & Woolhandler, S. (2014). Pay-for-performance: toxic to quality? Insights from behavioral economics. International Journal of Health Services: Planning, Administration, Evaluation, 44(2), 203–214.

Holcombe, R. F. (2014). Cancer clinical research: return on investment in the era of value-based purchasing. Journal of Oncology Practice/American Society of Clinical Oncology, 10(5), 327–328. http://doi.org/10.1200/JOP.2014.001416

Hupp, J. R. (2014). The value of improving value. Journal of Oral and Maxillofacial Surgery: Official Journal of the American Association of Oral and Maxillofacial Surgeons, 72(5), 843–845. http://doi.org/10.1016/j.joms.2014.03.001

Ireland, M., Paul, E., & Dujardin, B. (2011). Can performance-based financing be used to reform health systems in developing countries? Bull World Health Organ, pp. 695–698.

Jeong, H.-S. (2012). Designing an Effective Pay-for-performance System in the Korean National Health Insurance, Designing an Effective Pay-for-performance System in the Korean National Health Insurance. Journal of Preventive Medicine and Public Health, Journal of Preventive Medicine and Public Health, 45(3), 127–136. http://dx.doi.org/10.3961/jpmph.2012.45.3.127

Johnson, J., & Higgins, A. (2014). Evaluating the fair market value of pay for performance. Healthcare Financial Management: Journal of the Healthcare Financial Management Association, 68(4), 80–84.

Karash, J. A. (2013). Investing in value-based health care. Hospitals & Health Networks/AHA, 87(5), 54–58.

Lee, T.-T., Cheng, S.-H., Chen, C.-C., & Lai, M.-S. (2010). A pay-for-performance program for diabetes care in Taiwan: a preliminary assessment. The American Journal of Managed Care, 16(1), 65–69.

Lorincz, I. S., Lawson, B. C. T., & Long, J. A. (2013). Provider and patient directed financial incentives to improve care and outcomes for patients with diabetes. Current Diabetes Reports, 13(2), 188–195. http://doi.org/10.1007/s11892-012-0353-9

Maynard, A. D. (2011). The powers and pitfalls of payment for performance - Maynard - 2011 - Health Economics - Wiley Online Library. Health Economics, 21(1). http://onlinelibrary.wiley.com/doi/10.1002/hec.1810/abstract

McMahon, L. F., & Chopra, V. (2012). Health care cost and value: the way forward. Journal of the American Medical Association, 307(7), 671–672. http://doi.org/10.1001/jama.2012.136

Moore, L. G., & DeBuono, B. (2013). Total Cost of Care: A Discipline That Leads to Better Care. Journal of Ambulatory Care Management, 36(3), 193–198. http://doi.org/10.1097/JAC.0b013e3182955b4b

Peabody, J. W., Florentino, J., Shimkhada, R., Solon, O., & Quimbo, S. (2010). Quality variation and its impact on costs and satisfaction: evidence from the QIDS study. Medical Care, 48(1), 25–30. http://doi.org/10.1097/MLR.0b013e3181bd47b2

Rajkumar, R., Conway, P. H., & Tavenner, M. (2014). CMS--engaging multiple payers in payment reform. Journal of the American Medical Association, 311(19), 1967–1968. http://doi.org/10.1001/jama.2014.3703

Ran, L., Luo, K., Wu, Y., Yao, L., & Feng, Y. (2013). An analysis of China’s physician salary payment system. Journal of Huazhong University of Science and Technology. 33(2), 309–314. http://doi.org/10.1007/s11596-013-1116-9

Robeznieks, A. (2012). Looking at value. NQF backs effort focusing on resource use, costs. Modern Healthcare, 42(6), 10.

Rosenau, P. V., Lal, L. S., & Lako, C. (2012). Managing pay for performance: aligning social science research with budget predictability. Journal of Healthcare Management/American College of Healthcare Executives, 57(6), 391–404; discussion 404–405.

Ryan, A. M. (2013). Will value-based purchasing increase disparities in care? The New England Journal of Medicine, 369(26), 2472–2474. http://doi.org/10.1056/NEJMp1312654

Saronga, H. P., Duysburgh, E., Massawe, S., Dalaba, M. A., Savadogo, G., Tonchev, P., … Loukanova, S. (2014). Efficiency of antenatal care and childbirth services in selected primary health care facilities in rural Tanzania: a cross-sectional study. BMC Health Services Research, 14, 96. http://doi.org/10.1186/1472-6963-14-96

Swensen, S. J., Dilling, J. A., Mc Carty, P. M., Bolton, J. W., & Harper, C. M. (2013). The business case for health-care quality improvement. Journal of Patient Safety, 9(1), 44–52. http://doi.org/10.1097/PTS.0b013e3182753e33

Tan, E. C.-H., Pwu, R.-F., Chen, D.-R., & Yang, M.-C. (2014). Is a diabetes pay-for-performance program cost-effective under the National Health Insurance in Taiwan? Quality of Life Research: An International Journal of Quality of Life Aspects of Treatment, Care and Rehabilitation, 23(2), 687–696. http://doi.org/10.1007/s11136-013-0502-x

Tummers, J. F. M. M., Schrijvers, A. J. P., & Visser-Meily, J. M. A. (2013). A qualitative study of stakeholder views on the effects of provider payment on cooperation, quality of care and cost-containment in integrated stroke care. BMC Health Services Research, 13, 127. http://doi.org/10.1186/1472-6963-13-127

VanLare, J. M., Blum, J. D., & Conway, P. H. (2012). Linking performance with payment: implementing the Physician Value-Based Payment Modifier. Journal of the American Medical Association, 308(20), 2089–2090. http://doi.org/10.1001/jama.2012.14834

Wilson, K. J. (2013). Pay-for-performance in health care: what can we learn from international experience? Quality Management in Health Care, 22(1), 2–15. http://doi.org/10.1097/QMH.0b013e31827dea50

Wranik, D. (2012). Healthcare policy tools as determinants of health-system efficiency: evidence from the OECD. Health Economics, Policy, and Law, 7(2), 197–226. http://doi.org/10.1017/S1744133111000211

Yip, W., Powell-Jackson, T., Chen, W., Hu, M., Fe, E., Hu, M., … Hsiao, W. C. (2014). Capitation combined with pay-for-performance improves antibiotic prescribing practices in rural China. Health Affairs (Project Hope), 33(3), 502–510. http://doi.org/10.1377/hlthaff.2013.0702

Zeng, W., Rwiyereka, A. K., Amico, P. R., Avila-Figueroa, C., & Shepard, D. S. (2014). Efficiency of HIV/AIDS health centers and effect of community-based health insurance and performance-based financing on HIV/AIDS service delivery in Rwanda. The American Journal of Tropical Medicine and Hygiene, 90(4), 740–746. http://doi.org/10.4269/ajtmh.12-0697

## Footnotes

1 It should be noted that a recent study published using the same data found that there were no measurable difference in estimated probability of reporting illness with diarrhea, fever or acute respiratory infections between the intervention and comparison groups [28].
